# Long-term outcome after decompressive hemicraniectomy for malignant middle cerebral artery infarction

**DOI:** 10.1007/s00415-023-11679-1

**Published:** 2023-04-01

**Authors:** Natalie Berger, Anna Brunner, Gerit Wünsch, Oliver Nistl, Daniela Pinter, Simon Fandler-Höfler, Melanie Haidegger, Alexander Pichler, Isra Hatab, Michael Mokry, Stefan Wolfsberger, Christian Enzinger, Thomas Gattringer, Markus Kneihsl

**Affiliations:** 1 Department of Neurology, Medical University of Graz, Graz, Austria; 2 Department of Neurosurgery, Medical University of Graz, Graz, Austria; 3 Institute for Medical Informatics, Statistics and Documentation, Medical University of Graz, Graz, Austria; 4 Division of Neuroradiology, Department of Radiology, Medical University of Graz, Graz, Austria

**Keywords:** Ischemic stroke, Decompressive hemicraniectomy, Long-term outcome, Quality of life, Rehabilitation

## Abstract

**Background:**

Although decompressive hemicraniectomy (DHC) is a lifesaving treatment strategy for patients with malignant middle cerebral artery infarction (mMCAi), only one in four patients achieves low to moderate post-stroke disability according to previous studies. However, the short follow-up periods in prior studies could have overestimated the poor clinical prognosis. This study therefore examined the long-term outcome after DHC for mMCAi.

**Methods:**

We retrospectively included all patients who had undergone DHC after mMCAi at the University Hospital Graz between 2006 and 2019. Demographics, clinical data and complications were collected from electronic clinical patient records. To investigate long-term prognosis, all patients were followed up to 14 years after stroke including quality of life (QOL) assessment. Post-stroke disability was rated according to the modified Rankin Scale (mRS).

**Results:**

Of 47 patients that had undergone DHC for mMCAi, follow-up data were available in 40 patients (mean age: 48 years; 40% female). Six months after the mMCAi, 14 patients had died (35%) and nine (23%) had a low to moderate post-stroke disability (mRS 0–3). Of 26 stroke survivors, half (50%) showed further mRS improvement (≥ 1 point) during the long-term follow-up period (mean follow-up time: 8 years). At last follow-up, 17 patients had achieved an mRS score of ≤ 3 (65% versus 35% after 6 months; *p* = 0.008) and 55% had no signs of depression and anxiety, and 50% no signs of pain or discomfort in QOL assessment.

**Conclusion:**

This study shows substantial long-term improvement of functional disability and reasonable QOL in mMCAi patients after DHC.

## Introduction

Stroke is the second-leading cause of death globally and the leading cause of serious adult disability in industrial countries (1–3). Malignant middle cerebral artery infarction (mMCAi) is a feared complication of large-volume ischemic strokes in the anterior circulation especially in younger patients and the most common cause of death within 7 days after stroke [4].

Despite substantial developments in acute stroke care over the past years (such as mechanical thrombectomy), recent studies have shown that the rate of mMCAi’s remains high [5].

In this context, decompressive hemicraniectomy (DHC) can be a life-saving treatment strategy that was shown to greatly reduce mortality rates and to increase favorable outcomes according to modified Rankin Scale (mRS) scores of 0–3 [6, 7]. However, reduction in mortality also markedly increased the number of patients with severe persistent deficits (mRS scores 4–5) in clinical trials [8]. It has therefore been implicated that DHC might lead to survival at the cost of poor quality of life (QOL) in a significant number of mMCAi patients which affects preoperative benefit-risk assessment [9].

As an important limitation regarding the validity of these conclusions, the relatively short follow-up periods (mostly 6–12 months) in the randomized controlled trials could have overestimated the rate of poor functional outcome after DHC [6, 7, 10–12]. There is also limited data on QOL assessments in patients after mMCAi. This prompted us to follow mMCAi patients via a clinical long-term follow-up and to assess the evolution of functional disability and QOL.

## Methods

### Study design and data collection

We retrospectively evaluated all patients who had undergone DHC for mMCAi at the University Hospital Graz between 2006 and 2019. For data acquisition, we used the hospital information system *medical and nursing documentation and communication network of Styria* (MEDOCS), a system that covers medical information collected in all public hospitals in the province of Styria, where the University Hospital is the single center offering DHC [13]. All study participants were reviewed for demographics, vascular risk factors, previous vascular events, initial clinical symptoms, data concerning recanalization therapies and imaging parameters.

#### Decompressive hemicraniectomy

DHC was performed by experienced neurosurgeons according to latest guideline recommendations [14]. Therefore, DHC was usually performed in patients ≤ 60 years of age and within 48 h after symptom onset. In individual cases, (e.g., excellent prehospital health status) patients above an age of 60 years were also treated with DHC as were patients > 48 h after symptom onset in case of imminent herniation. Patients with severe preexistent disability or serious concomitant disease (e.g., terminal cancer) did not undergo DHC [14].

Anteroposterior length of DHC was aimed to be at least 12 cm. The extent of the removed bone flap was measured retrospectively by an experienced neuroradiologist (O.N.) using postoperative CT-imaging files. Furthermore, the final infarct size was calculated by analyzing the postoperative imaging.

After surgery, all patients underwent a standard treatment regime at the neurointensive care unit of our department.

We analyzed stroke- and surgery-associated complications, including pneumonia, herniation, complications associated with an installed external ventricular drainage (EVD), persistent hydrocephalus, infection of an installed ventriculoperitoneal shunt (VP-shunt) and clinically relevant intracranial bleeding (i.e. parenchymatous hematoma (PH) 1 and 2, defined by the *Heidelberg Bleeding Classification*) [15]. Furthermore, we analyzed mortality, cause of death and functional neurological outcome measured via the mRS 3 and 6 months after stroke. Follow-up examinations including mRS ratings were conducted by experienced neurologists in clinical face-to-face visits.

#### Long-term follow-up

We captured long-term follow-up data via structured telephone visits either with the patients themselves or with their next of kin. We determined the current mRS and gathered information on duration and time of neurological rehabilitation [16, 17]. Furthermore, we conducted a depression and anxiety assessment using the Hospital Anxiety and Depression Scale HADS [18]. HADS consists of two subscales, seven questions each, related to anxiety or depression (score range 0–21). Scores ≥ 8 have been reported to identify anxiety/depression [19].

We also collected data concerning patient’s health-related QOL using the five-dimension EuroQol (EQ-5D), a standardized instrument to measure five dimensions of health (*mobility, self-care, usual activities, pain and discomfort, anxiety and depression*) [20]. Each dimension is graded into three levels including *no problems*, *some problems* and *extreme problems*. Because of its simplicity, the EQ-5D can easily be conducted by phone or through caregivers. For further analyses, we dichotomized the answers into “no problems” and “problems” (including *some* and *extreme problems*) [21].

### Statistical analysis

Statistical analysis was performed using the IBM SSPSS Statistics version 27.0. For the description of the collected parameters, we used mean and standard deviation for continuous variables as well as median and range for ordinal data.

To compare categorical variables, we used the Chi-square test. Due to the small number of cases, comparisons between groups were made using Fisher’s exact analysis.

For continuous variables we tested Gaussian distribution with the Kolmogorov–Smirnov test. If Gaussian distribution was identified, we used the *T*-Test. For independent variables, the Mann–Whitney *U* Test was utilized. For subgroup analyses, we divided the included patients according to their mRS score 6 months after stroke (mRS score ≤ 3 versus mRS score > 3) and according to mRS improvement (mRS improvement ≥ 1 vs no mRS improvement) during the long-term follow-up period.

A *p* value less than 0.05 was considered statistically significant.

#### Ethics

The study protocol was approved by the local ethics committee. Informed consent was obtained from all included study participants.

## Results

**Fig. 1:**
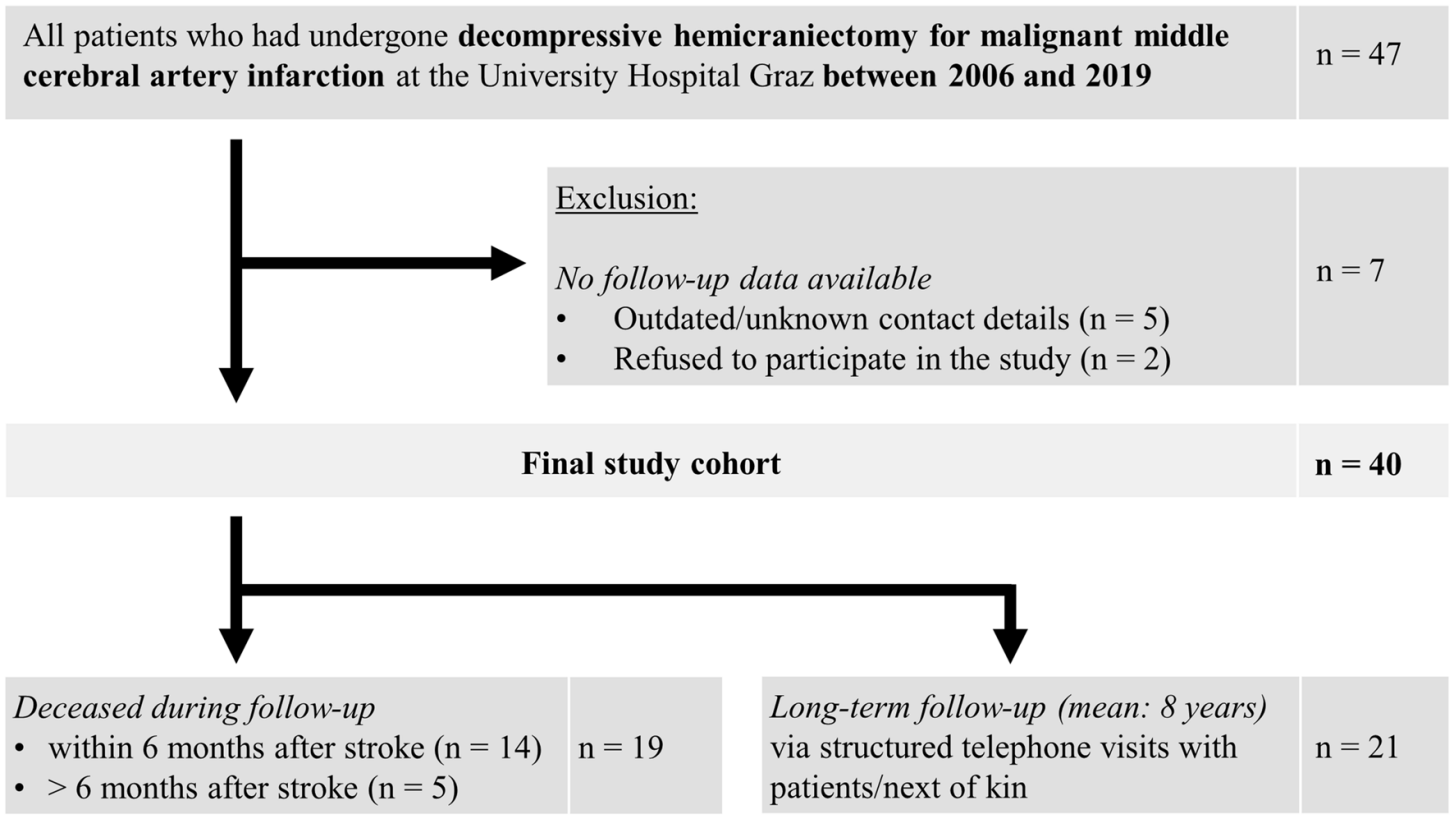
Study flow chart

Between September 2006 and April 2019, 47 patients had undergone DHC for mMCAi at the University Hospital in Graz. As seven patients had to be excluded due to missing (long-term) follow-up, the final study cohort comprised 40 patients (mean age 48 years, range 19–63 years; 40% female) (Fig. 1). All patients have had an intracranial large vessel occlusion causing the stroke (ICA 35%, MCA 65%), which was right-sided in 18 patients (45%). The most frequent vascular risk factors were obesity (75%), hyperlipidemia (70%) and arterial hypertension (60%). Half of patients had undergone an acute recanalization therapy to restore blood flow (intravenous thrombolysis [IVT]: 43%; mechanical thrombectomy [MT]: 13%). However, in only three patients, blood flow was successfully restored (15%).

IVT was withheld either because (1) of the extent of cerebral ischemia on initial brain imaging (30%), (2) passing of the therapeutic time window (28%) or (3) contraindications in terms of high bleeding risk (10%).

### DHC and peri/postoperative complications

DHC was performed at a mean time of 33 h after symptom onset/last seen well (range 10–91 h). Two patients (5%) had surgery > 60 h after symptoms onset.

The mean size of DHC was 127.3 cm^2^ (range 85–196 cm^2^). In five patients, the DHC diameter was < 12 cm (14%). Despite surgery, progressive brain herniation followed by death was observed in five patients (13%). A persistent hydrocephalus occurred in eleven patients (28%). Of those, five (13%) had to undergo further surgery for ventriculoperitoneal shunt implantation. Complications and baseline characteristics are presented in Table 1.

**Table 1:** Patients who underwent decompressive hemicraniectomy for malignant middle cerebral artery infraction between 2006 and 2019

Variable/clinical finding	DHC patients (*n* = 40)	mRS ≤ 3 at 6 months (*n* = 9)	mRS > 3 at 6 months (*n* = 31)	*p* value
Demographics/clinical parameters				
Age (years, mean, range)	48.2 (19–63)	39.5 (19–56)	49.8 (25–63)	0.020
Female (N, %)	16 (40)	4 (45)	12 (39)	1
Right-sided stroke (N, %)	18 (45)	3 (33)	15 (48)	0.476
Vascular risk factors				
Hypertension (N, %)	24 (60)	3 (33)	21 (68)	0.063
Obesity (N, %)	30 (75)	5 (55)	25 (81)	0.190
Hyperlipidemia (N, %)	28 (70)	5 (55)	23 (74)	0.411
Diabetes (N, %)	13 (33)	2 (22)	11 (35)	0.690
Nicotine abuse (N, %)	22 (55)	5 (55)	17 (55)	1
Admission NIHSS (median, SD)	17 (± 5)	19.5 (± 7)	16 (± 4)	0.319
IVT and/or MT (N, %)	20 (50)	5 (56)	15 (48)	1
Successful recanalization (N, %)	3 (8)	1 (11)	2 (6)	0.545
Surgery/imaging				
Duration symptom onset–DHC (h, mean, range)	34 (14.3–91.2)	34 (14.3–54.9)	34 (16.1–91.2)	0.937
Duration of DHC (h, mean, range)	1.5 (0.9–2.3)	1.5 (1.2–2.2)	1.5 (0.9–2.3)	0.131
DHC size (cm^2^, mean, range)	127 (96–168)	127 (96–154)	129 (102–168)	0.429
DHC < 12 cm diameter (N, %)	5 (13)	0	5 (16)	0.312
EVD (N, %)	25 (63)	6 (66)	19 (61)	1
Ventriculoperitoneal shunt (N, %)	5 (13)	1 (11)	4 (13)	1
Final infarct size (cm^2^, mean, range)	319 (140–605)	235 (140–350)	345 (208–605)	0.007
Complications and outcome				
Pneumonia (N, %)	23 (58)	7 (77)	16 (52)	0.256
Progressive herniation (N, %)	5 (13)	0	5 (16)	0.570
Ventriculitis (N, %)	2 (5)	0	2 (6)	1
Persistent hydrocephalus (N, %)	11 (28)	4 (44)	7 (23)	0.227
Ventriculoperitoneal shunt infection (N, %)	4 (10)	0	4 (13)	0.557
Intracerebral hemorrhage (N, %)	4 (10)	1 (11)	3 (10)	1
mRS at 6 months (median, range)	4 (± 1)	3 (± 0)	5 (± 1)	< 0.001

Demonstrated p value was determined by comparing patients with mRS < 3 to patients with mRS 4–6 six months after stroke

*NIHSS* National Institutes of Health Stroke Scale, *SD* standard deviation, *IVT* intravenous thrombolysis, *MT* mechanical thrombectomy, *DHC* decompressive hemicraniectomy, *EVD* external ventricular drainage

#### 6-months outcome

14 patients (35%) died within 6 months after stroke (mean time from symptom onset to death: 5 days; range 3–80 days). Except for mMCAi-related death (11 patients, 79%), two patients died from sepsis-induced organ failure (14%) and one due to pulmonary embolism (7%). Only nine patients (23%) reached an mRS of ≤ 3 6 months after stroke. Compared to patients with mRS 4–6 (*n* = 31, 77%), patients with favorable outcome were younger (mean age 40 vs. 50 years, *p* = 0.02) and had a smaller final infarct size (mean 244 vs. 290 ml, *p* = 0.004). Lateralization of infarction had no impact on the outcome at 6 months (right-sided stroke; 33 vs. 48%, *p* = 0.476) (Table 1).

#### Long-term FU and quality of live assessment

Patients underwent a mean follow-up of 8 years after DHC (range 4–14 years).

**Fig. 2:**
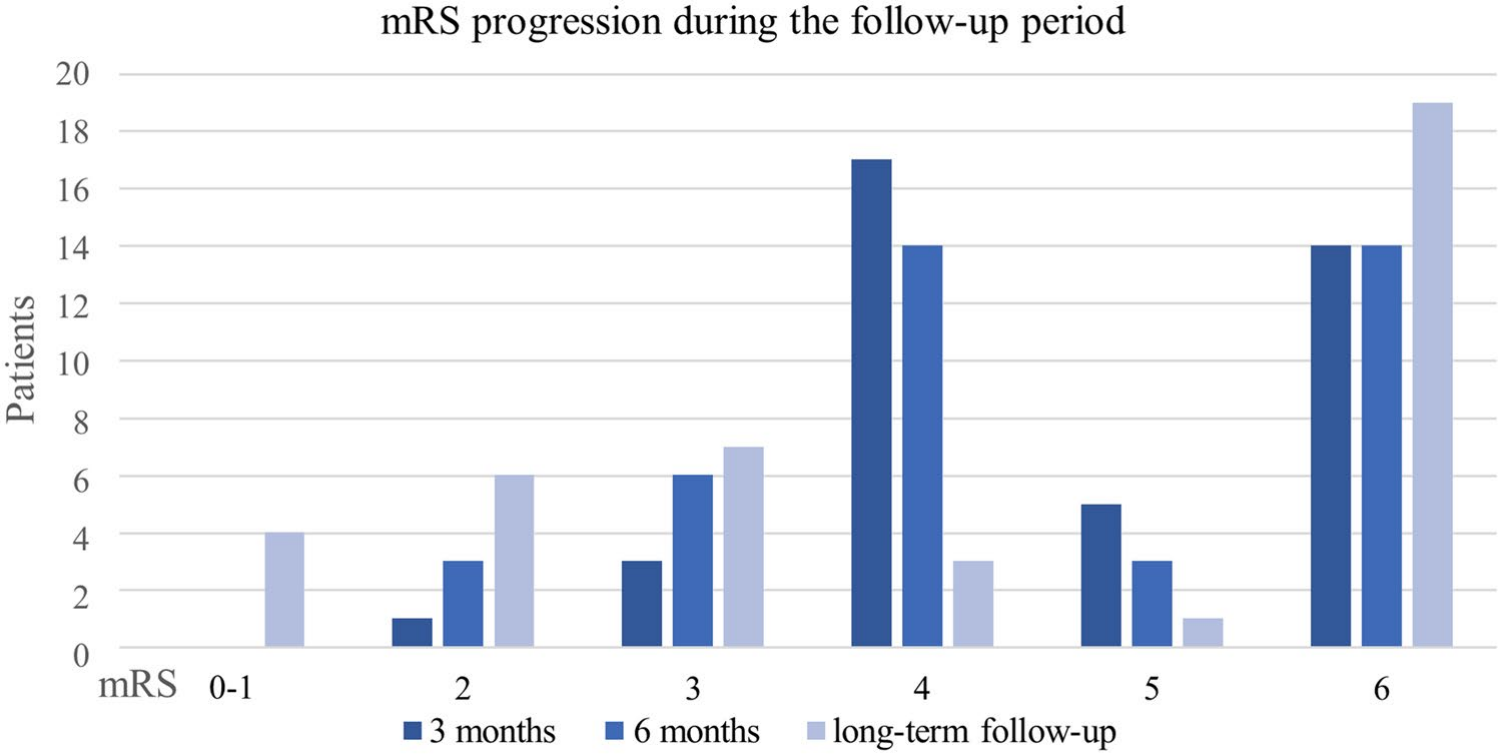
Long-term mRS progression of patients who had undergone decompressive hemicraniectomy for malignant middle cerebral artery infarction

At last follow-up, 17 patients (42%) achieved a favorable outcome according to an mRS score ≤ 3 compared to only 9 patients (22%) at 6 months. Of all 26 patients who survived the first 6-months period after stroke, we observed further mRS improvement of at least one point in 50% of patients (*n* = 13) during the long-term course (Fig. 2).

Patients with long-term mRS improvement (*n* = 13) tended to be younger (44 vs. 51 years, *p* = 0.127), were less often smokers (38% vs. 85%, *p* = 0.027) and received longer neurorehabilitation (24.9 vs. 11.8 weeks, *p* = 0.043) compared to patients who did not ameliorate from their 6-months disability level (Table 2).

**Table 2:** Clinical long-term improvement after decompressive hemicraniectomy in patients with malignant middle cerebral artery infarction

Variable/clinical finding	long-term mRS improvement (*n* = 13)	no long-term mRS improvement (*n* = 13)	*p* value
Demographics			
Age, years (mean, range)	44 (19–56)	51 (29–63)	0.127
Female (N, %)	6 (46)	5 (38)	1
Right-sided stroke (N, %)	6 (46)	3 (23)	0.411
Vascular risk factors			
Hypertension (N, %)	5 (38)	9 (69)	0.238
Obesity (N, %)	8 (62)	11 (85)	0.378
Hyperlipidemia (N, %)	7 (54)	11 (85)	0.202
Diabetes (N, %)	2 (15)	5 (38)	0.378
Nicotine abuse (N, %)	5 (38)	11 (85)	0.027
Admission NIHSS (median, SD)	17 (± 4)	17 (± 7)	0.786
IVT and/or MT (N, %)	7 (54)	7 (54)	1
Successful recanalization (N, %)	2 (15)	0	0.200
Surgery/imaging			
Duration symptom onset–DHC (h, mean, range)	39.7 (16.1–91.2)	27.5 (14.3–49.1)	0.098
Duration of DHC (h, mean, range)	1.5 (0.9–2.3)	1.5 (1.1–2.2)	0.653
DHC size (cm^2^, mean, range)	132 (108–168)	126 (96–147)	0.396
DHC < 12 cm diameter (N, %)	0	2 (15)	0.480
EVD (N, %)	6 (46)	8 (62)	0.695
Ventriculoperitoneal shunt (N, %)	2 (15)	2 (15)	1
Final infarct size (cm^2^, mean, range)	248 (202–301)	280 (140–420)	0.241
Complications and outcome			
Pneumonia (N, %)	8 (62)	9 (69)	1
Ventriculitis (N, %)	1 (8)	1 (8)	1
Persistent Hydrocephalus (N, %)	4 (31)	4 (31)	1
Ventriculoperitoneal shunt infection (N, %)	2 (15)	1 (8)	0.400
Intracerebral hemorrhage (N, %)	1 (8)	2 (15)	1
mRS at 6 months (median, SD)	4 (± 0.8)	4 (± 0.9)	0.531
mRS at last follow-up (median, SD)	2 (± 1)	4 (± 1.4)	< 0.001
Duration of rehabilitation after 6 months (mean, range)	24.9 (3–56)	11.8 (2–31)	0.043

Demonstrated *p* value was determined by comparing patients with long-term mRS improvement to patients with no long-term mRS improvement

*NIHSS* National Institutes of Health Stroke Scale, *SD* standard deviation, *IVT* intravenous thrombolysis, *MT* mechanical thrombectomy, *DHC* decompressive hemicraniectomy, *EVD* external ventricular drainage

Of note, six of eight patients (75%) under the age of 40 achieved a favorable outcome.

**Fig. 3:**
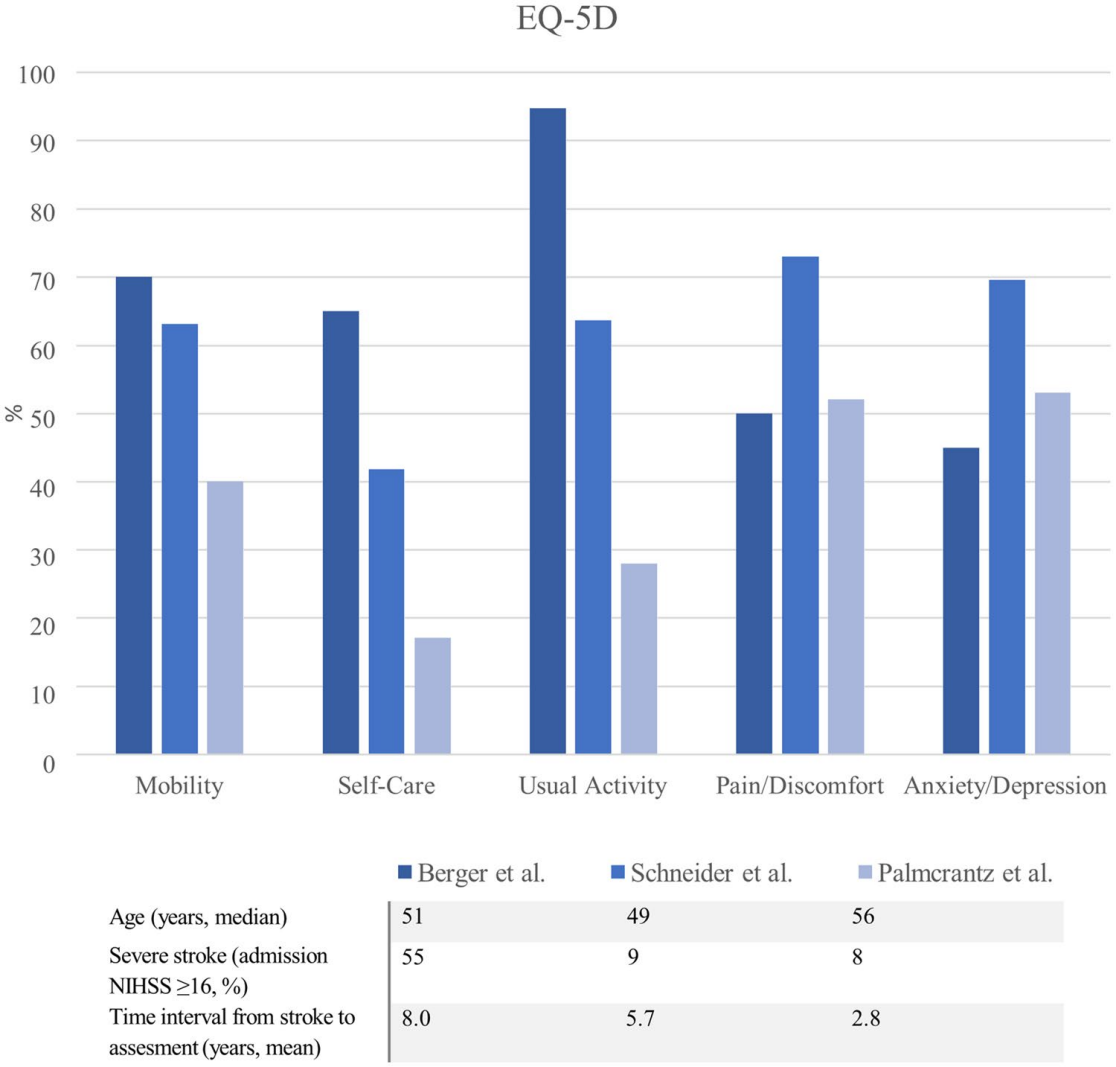
Comparison of long-term quality of live follow-ups via the EQ-5D assessment in cohorts of young stroke patients

EQ-5D assessment showed that *usual activity* (95%), mobility (70%) and self-care (65%) remained substantially affected in most mMCAi patients at long-term follow-up. However, only 45% of patients reported at least moderate problems in the section *anxiety and depression* and 50% for *pain and discomfort*. Figure 3 shows a comparison of our results with those of other studies analyzing long-term QOL using the EQ-5D assessment in young stroke patients [21, 22].

## Discussion

To the best of our knowledge, we here present the longest follow-up study of patients who had undergone DHC for mMCAi and identified clinical mRS score improvement in every second stroke survivor beyond the standard 6-months outcome assessment. The rate of patients with a favorable outcome (mRS 0–3) doubled from 22% at 6 months to 42% at the last follow-up (mean: 8 years post stroke).

Our results are of clinical interest as benefit-risk assessment for DHC in patients with mMCAi is strongly influenced by outcome values. However, the endpoints of the randomized controlled trials were predominantly set at 6–12 months post stroke [6, 7, 10–12, 23].

From clinical observations and based on recent study results showing long-term cognitive improvement after first ever stroke in young patients, [24] previous studies thus might have underestimated the number of patients with favorable clinical outcome after mMCAi.

To date, a follow-up investigation of patients randomized in the HAMLET trial was the only study that analyzed mRS scores > 12 months after decompressive hemicraniectomy for mMCAi. However, Geurts and colleagues could not identify long-term mRS improvement compared to their 6–12 months outcome [25]. These differences to our findings might be explained by (1) the shorter follow-up duration in the study of Geurts and colleagues (three vs. eight years) and (2) the extensive duration of rehabilitation in survivors with mRS improvement in our cohort (mean: 6 months) [25]. As the potential for rehabilitation success and neuroplasticity are related to age, it is not surprising that patients with a favorable long-term prognosis after DHC for mMCAi were approximately 10 years younger than those with long-term mRS 4–6. Of note, 80% of patients under the age of 40 achieved a favorable outcome, representing a specific subgroup with good long-term prognosis.

In this study, we could not identify a significant influence of lateralization of infarction on outcome after DHC neither at the 6-months nor at the long-term endpoint. This supports the conclusion of a recent review by Lin et al., who also stated that functional outcome of patients who had undergone DHC for mMCAi was not affected by lateralization of the infarction (dominant vs non-dominant hemisphere) [26].

A further strength of our study is that we had QOL measures at the last follow-up available. As we here report findings from a cohort of severely affected stroke patients, our EQ-5D results of persistent deficits in *usual activity, mobility* and *self-care* are not surprising. However, we found satisfactory results in the domains *anxiety and depression* as well as *pain and discomfort* as more than half of all patients (~ 60%) had no long-term problems in these sections. Moreover, only one-third of patients showed a post-stroke depression according to HADS. Although a strong correlation between stroke severity and post-stroke depression/anxiety has been reported, [27] the long-term depression/anxiety rates in our study population are comparable or even lower to those of recent studies in general young stroke patients [21, 22, 28–30]. Our results therefore indicate that the majority of mMCAi survivors achieves a satisfying level of daily life well-being.

The current study also has some limitations. First, baseline data were retrospectively collected from clinical patient records that were originally not designed to answer the given study question. Second, this is a single-center study with a relatively small study cohort that precluded multivariable analysis of outcome measures. Nevertheless, we here present real-world follow-up data of 40 DHC patients after mMCAi, which is an even larger cohort compared to the only available long-term follow-up study of 32 patients receiving DHC in the HAMLET trial [26].

A further limitation was that the follow-up had to be conducted by phone due to Covid-19 restrictions. However, numerous studies showed good agreement between functional outcome/QOL assessments via personal face-to-face visits and structured telephone interviews [16, 17].

## Conclusions

In conclusion, this single-center study in real-world patients shows substantial long-term improvement of functional disability and moderate rates of depression and/or anxiety in patients who had undergone DHC for mMCAi. Although our findings need to be replicated in larger (multicenter) studies or pooled analysis of existing cohorts, they might be considered in preoperative benefit-risk assessments.
